# IL-6R Inhibitors and Gastrointestinal Perforations: A Pharmacovigilance Study and a Predicting Nomogram

**DOI:** 10.3390/biomedicines12122860

**Published:** 2024-12-17

**Authors:** Shupeng Zou, Mengling Ouyang, Qian Cheng, Xuan Shi, Yazheng Zhao, Minghui Sun

**Affiliations:** Department of Pharmacy, Tongji Hospital, Tongji Medical College, Huazhong University of Science and Technology, Wuhan 430030, China; tjzoushupeng@tjh.tjmu.edu.cn (S.Z.); oyml@hust.edu.cn (M.O.); chengqian0713@tjh.tjmu.edu.cn (Q.C.); xuanshi@tjh.tjmu.edu.cn (X.S.); yazh_zhao@tjh.tjmu.edu.cn (Y.Z.)

**Keywords:** signal detection, IL-6 inhibitors, tocilizumab, intestinal perforation, pharmacovigilance

## Abstract

**Objective** IL-6R inhibitors are widely used in many inflammation-related diseases, especially so during the COVID-19 pandemic. However, their relationship with gastrointestinal perforations (GIPs) has been reported more and more. We comprehensively analyzed IL-6R inhibitors in association with GIPs from the United States FDA Adverse Event Reporting System (FAERS). **Methods:** A disproportionate analysis was used to quantify the signals of GIPs caused by IL-6R inhibitors using two algorithms, and we assessed the risk using logistic regression analysis. We also established a risk prediction model of GIPs. **Results:** We identified 994 cases with GIPs of IL-6R inhibitors (tocilizumab and sarilumab) from the FAERS database. The GIPs signals of IL-6R inhibitors were significant, including tocilizumab (reporting odds ratio [ROR] 6.86, 95%CI 6.43–7.31) and sarilumab (ROR 4.03, 95%CI 2.83–5.73). Duodenal perforation had the strongest signals of tocilizumab (*n* = 312; ROR 19.45, 95%CI 17.33–21.83; IC_025_ 3.72) and sarilumab (*n* = 14; ROR 9.57, 95%CI 5.66–16.17; IC_025_ 1.92). The median time to GIPs was near 60 days. In total, 71% of the cases occurred within the first six months after tocilizumab treatment. After excluding missing data, we found that independent risk factors included female (OR 1.52, 95%CI 1.16–1.98), ≥40 years (OR 5.63, 95%CI 1.78–17.78), glucocorticoids (OR 1.37, 95%CI 1.10–1.72), and nonsteroidal anti-inflammatory drugs (NSAIDs, OR 3.46, 95%CI 2.77–4.32). The risk prediction model showed good discrimination and clinical applicability in both the training (AUC, 0.73) and validation (AUC, 0.75) sets. **Conclusions:** IL-6R inhibitors may increase the risk of GIPs, especially female, middle-aged patients, IL-6R inhibitors, NSAIDs, and glucocorticoids. Therefore, we suggest that these factors associated with gastrointestinal reactions should be considered during treatment.

## 1. Introduction

Humanized anti-interleukin-6 receptor (IL-6R) monoclonal antibodies are used in many inflammation-related diseases, such as active rheumatoid arthritis (RA), giant cell arteritis (GCA), cytokine release syndrome (CRS), and coronavirus disease 2019 (COVID-19) [1,2,3]. Interleukin-6 (IL-6), which induces B cells to mature into immunoglobulin-secreting plasma cells, is closely associated with immune regulation and dysregulation in several diseases [4,5]. In COVID-19, the Australian guidelines suggest that tocilizumab should be reserved for patients/children with evidence of systemic inflammation [6]. Meanwhile, gastrointestinal perforations caused by tocilizumab were increasingly reported [7,8,9,10]. Our study analyzed the relationship between IL-6R inhibitors and gastrointestinal perforations comprehensively and established a nomogram to predict gastrointestinal perforations.

At present, many IL-6R inhibitors are on the market, such as siltuximab, clazakizumab, olokizumab, satralizumab, sarilumab, and tocilizumab. Because of fewer cases of other IL-6R inhibitors in FAERS, we only studied the cases of tocilizumab and sarilumab. Tocilizumab has also been used to treat immune-related adverse events (AEs) induced by other therapies. In a consensus report of the European Myeloma Network, premedication with tocilizumab reduced CRS associated with CAR-T cells in multiple myeloma [11]. In a review of four cases, tocilizumab appeared to be a promising agent for steroid-refractory immune-related hepatotoxicity (IRH) including immune checkpoint inhibitors (ICIs) [12]. In a consensus statement regarding IL-6 inhibitors in immune-mediated inflammatory diseases (including COVID-19, Takayasu arteritis, and Still’s disease), the safety outcomes of tocilizumab did not differ from those of other biological disease-modifying antirheumatic drugs (DMARDs), except for higher risks of diverticulitis and lower GIPs [13].

Inevitably, the adverse events associated with tocilizumab, particularly serious infections and cardiovascular events, should not be ignored [8]. Gastrointestinal (GI) perforations are also concerning. Our study found that the number of GIPs cases associated with IL-6R inhibitors (tocilizumab and sarilumab) increased from 2019 to 2023. A population-based study (French) on the risk of GI perforation in RA treated with tocilizumab showed that the risk of GI perforation and diverticulitis could be increased in 1196 patients receiving tocilizumab [9]. In a nationwide cohort study (Sweden) of 63,532 patients with RA, tocilizumab (adjusted HR 2.2, 95%CI 1.3–3.8) was associated with a higher risk of lower GI perforations than tumor necrosis factor inhibitors (TNFi), after adjusting for all confounders by the inverse probability of treatment weighting (IPTW) [7]. In an observational study (German) of 13,310 patients with RA, the incidence rates of lower intestinal perforations caused by tocilizumab were similar to those of the randomized controlled trials of tocilizumab and were higher than those of all other DMARDs [14]. As early as 2011, a systematic literature review reported that, while tocilizumab treatment might marginally elevate the risk of diverticular perforation compared to traditional DMARDs or anti-TNF agents, this risk was still lower than that of corticosteroid treatments [15]. Moreover, the lethality of GI perforations is high (approximately 30%) and increases with age [16,17]. 

The exact mechanism of GI perforation induced by tocilizumab is unclear and is likely multifactorial. However, nonsteroidal anti-inflammatory drugs (NSAIDs) are associated with an increased risk of GI bleeding, perforation, and ulceration. NSAIDs potentially contribute to intestinal perforation [18]. Another explanation for the biological mechanisms is that IL-6 expression is observed early after GI injury, in which IL-6 regulates vascular endothelial growth to support epithelial proliferation and wound healing [7,19,20]. Although it has been reported that tocilizumab increases the risk of gastrointestinal perforation, the relationship between sarilumab and gastrointestinal perforations is not clear. In addition, there are no studies on predictive models about anti-IL-6R monoclonal antibody and GI perforations.

Our retrospective study firstly used the disproportionate method and logistic regression analysis to evaluate the risk of GI perforation associated with IL-6R inhibitors from the FAERS database. We also established a nomogram model to predict gastrointestinal perforations in patients with IL-6R inhibitors.

## 2. Results

### 2.1. Descriptive Analysis

**Figure 1:**
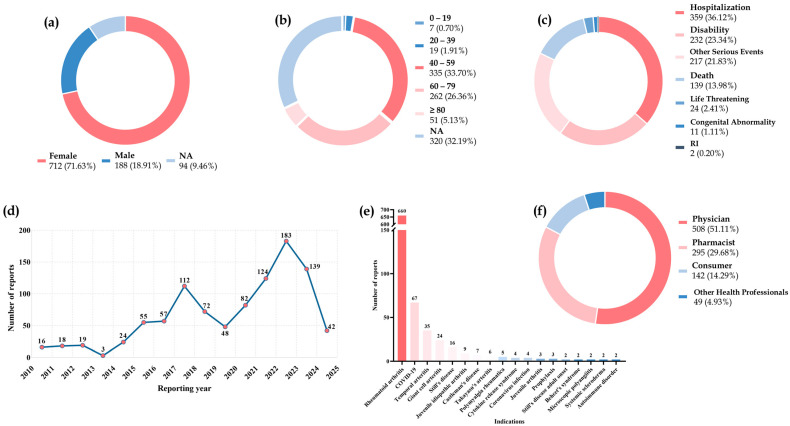
The clinical characteristics of patients with IL-6R inhibitors-induced gastrointestinal perforations in the FAERS database from 2010 Q1 to 2024 Q1. (**a**) The gender distribution of patients. (**b**) The age distribution of patients. (**c**) The serious outcome distribution of adverse reactions in patients. (**d**) The number of annual adverse reaction reports. (**e**) The indications of patients. (**f**) The occupational distribution of the reporter. FAERS: food and drug administration adverse event reporting database. NA: unknown. Q: quarter. RI: required intervention to prevent permanent impairment/damage.

The related data distribution of GI perforations after data mining from 2010 (Q1, the first quarter) to 2024 (Q1) is shown in Figure 1. Overall, the number of female patients with GI perforations (*n* = 712, 71.63%) was larger than that of male patients (*n* = 188, 18.91%). The median (IQR) age of the patients with GI perforations was 54 (44–68) years, mainly 40–59 years (*n* = 335, 33.70%) and 60–79 years (*n* = 262, 26.36%). Among the outcomes of GI perforations, hospitalization (*n* = 359, 36.12%) was the most commonly reported event, followed by disability (*n* = 232, 23.34%), which required special attention. During the reporting period from 2019 to 2023, there was an increase in GI perforations coinciding with the COVID-19 pandemic. Among the indications for tocilizumab for GI perforations, RA (*n* = 660, 66.40%) was the most frequently reported, followed by COVID-19 (67, 6.74%), temporal arteritis (35, 3.52%), and giant cell arteritis (24, 2.41%).

### 2.2. Perforation Location Descriptions

**Figure 2:**
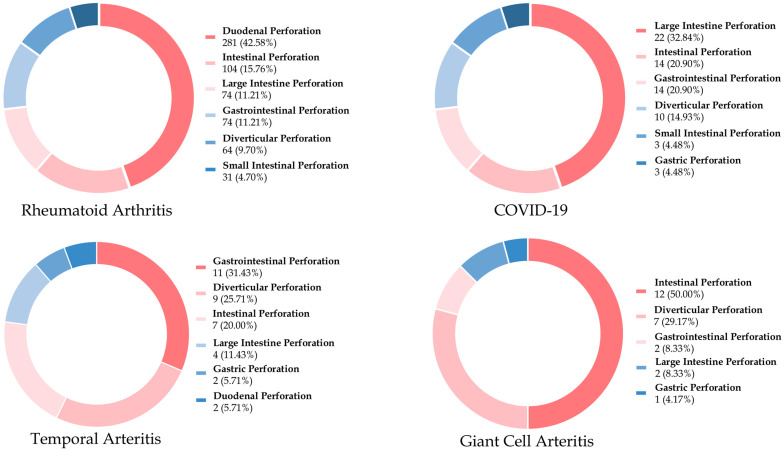
The top six locations of gastrointestinal perforations associated with the top four indications of IL-6R inhibitors from the FAERS database.

In the top four indications for IL-6R inhibitors, we classified the perforation location as gastroesophageal, small intestinal, intestinal, gastrointestinal, large intestine, diverticular, and gastric perforation [21]. In Figure 2, the most common location was duodenal perforation (*n* = 281, 42.58%) in RA, followed by COVID-19 (large intestine perforation; *n* = 22, 32.84%), temporal arteritis (gastrointestinal perforation; *n* = 11, 31.43%) and giant cell arteritis (intestinal perforation; *n* = 12, 50%).

### 2.3. Disproportionality Analysis

As shown in Table 1, we used two algorithms to evaluate the association between GI perforation and IL-6R inhibitors. The signals of the GI perforations (all) of tocilizumab and sarilumab showed significance, as tocilizumab (*n* = 963; ROR 6.86, 95%CI 6.43–7.31; IC_025_ 2.57) and sarilumab (*n* = 31; ROR 4.03, 95%CI 2.83–5.73; IC_025_ 1.41), respectively. Duodenal perforation showed the strongest correlation with tocilizumab (*n* = 312; ROR 19.45, 95%CI 17.33–21.83; IC_025_ 3.72) or sarilumab (*n* = 14; ROR 9.57, 95%CI 5.66–16.17; IC_025_ 1.92). IC_025_ > 3.0, it indicated a strong signal; 1.5 < IC_025_ ≤ 3.0, it indicated a medium-intensity signal [22]. The majority of GI perforations were significant in IL-6R inhibitors. Notably, duodenal perforation, large intestine perforation, and diverticular perforation showed non-significant difference in tocilizumab and sarilumab by Pearson chi-square test (*p* > 0.05). But in total, GI perforations (all) showed significant difference in tocilizumab and sarilumab (*p* < 0.001).

### 2.4. Stratification Analysis

**Figure 3:**
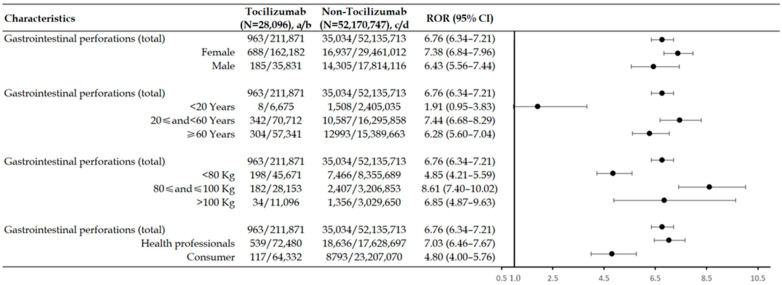
Stratification analysis of tocilizumab-related gastrointestinal perforations. N, number of cases of total AEs associated with the target drug; ROR, reporting odds ratio; a/c, number of cases with suspected AEs associated with the target drug; b/d, number of cases without suspected AEs (i.e., total AEs excluding suspected ones) associated with the target drug. The forest plot from the stratification analysis was based on ROR values, indicating that a higher value was associated with a stronger correlation.

We conducted a stratification analysis of GI perforations (total) of tocilizumab in Figure 3 and used four different layering strategies (sex, age, weight, and reporters) to analyze the association between GI perforations and tocilizumab. After dividing GI perforations (total) by sex, age, body weight, and reporter type, the lower limit of the ROR in all stratified subgroups was greater than 1, indicating that there was still a strong statistical correlation between tocilizumab and GI perforations. Interestingly, the ROR (1.91, 95%CI 0.95–3.83) of the age group (<20 years) showed a non-significant difference. In the age and weight groups, we found that ROR values also increased with age (<60 years) and weight (≤100 kg). Generally, the highest ROR in the different subgroups were as follows: female (ROR 7.38, 95%CI 6.84–7.96), 20 ≤ and < 60 years (ROR 7.44, 95%CI 6.68–8.29), 80 ≤ and ≤ 100 kg (ROR 8.61, 95%CI 7.40–10.02), and health professionals (ROR 7.03, 95%CI 6.46–7.67).

### 2.5. Time-to-Onset Analysis

We collected the onset time of IL-6R inhibitors-related GI perforations (231 cases) from the FAERS database, and the results of the time to onset (TTO) and the Weibull shape parameter (WSP) analysis were shown in Supplementary Table S1. The median TTO of IL-6R inhibitors was 60 (IQR [interquartile range], 13–239) days of tocilizumab and 79 (IQR 13–202) days of sarilumab, with approximately 71% of cases occurring within the first six months after starting tocilizumab. According to the WSP analysis, the shape parameter β and its upper limit of 95% CI were both <1, suggesting that tocilizumab was early type of failure and sarilumab was random failure in GI perforations.

### 2.6. Analysis of Risk Factors

After excluding missing data, we used univariate and multivariable logistic regression analyses to evaluate the risk factors for GI perforations, including sex, age, weight, NSAIDs, glucocorticoids, methotrexate (MTX), and IL-6R inhibitors. As shown in Table 2, compared with the reference in different groups, we found that some factors were at high risk, including female (OR 1.52, 95%CI 1.16–1.98), ≥40 years (OR 5.63, 95%CI 1.78–17.78), tocilizumab (OR 2.94, 95%CI 1.50–5.79), glucocorticoids (OR 1.37, 95%CI 1.10–1.72), and NSAIDs (OR 3.46, 95%CI 2.77–4.32). 

### 2.7. A Predicting Nomogram and Validation

**Figure 4:**
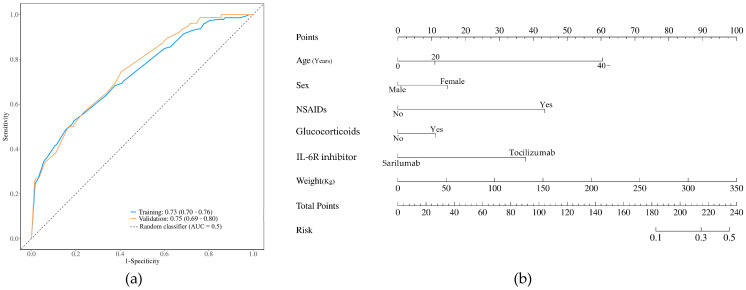
A nomogram model for predicting gastrointestinal perforations in patients with IL-6R inhibitors. (**a**) A receiver operating characteristic (ROC) curve analysis. (**b**) A predicting nomogram of gastrointestinal perforations. NSAIDs: nonsteroidal anti-inflammatory drugs. AUC: area under curve.

After excluding missing data and using a stepwise backward regression method, data from 9717 patients treated with IL-6R inhibitors were extracted, including information on sex, age, weight, and concomitant medications (NSAIDs or glucocorticoids). The included cases were randomly divided 8:2 into the training and validation sets. After the logistic regression analysis, in Figure 4a, the resulting receiver operating characteristic (ROC) curve analysis showed that the area under curve (AUC) of the training set and that of the validation set were 0.73 (0.70–0.76) and 0.75 (0.69–0.80), showing good model discrimination [23]. The Hosmer–Lemeshow test (*p* = 0.087) showed a good overall fit. The risk factors for GI perforation were used to construct a nomogram model, as shown in Figure 4b. For example, a 40-year and 80 kg female treated with concomitant medications (NSAIDs, glucocorticoids, and tocilizumab) scored 192 points approximately, corresponding to a near 12% risk of gastrointestinal perforations.

## 3. Discussion

We systematically and comprehensively evaluated GI perforations associated with IL-6R inhibitors using the FAERS database and plotted a predictive nomogram. In a study of IL-6R inhibitors (tocilizumab and sarilumab) from the FAERS database, there was a strong correlation with gastrointestinal issues, except for hypercholesterolemia, severe infections, diabetes, and nervous system disorders [24]. A long-term safety study of tocilizumab showed that the overall incidence of GI perforation during follow-up was 0.65% [25]. A previous study reported an increased mortality risk following lower GI perforation in patients treated with tocilizumab [14]. GI perforations are costly, severe, and potentially life threatening. A long-term (7-year follow-up) safety of sarilumab in rheumatoid arthritis showed 0.1% for gastrointestinal perforations in the combination group [26]. Another long-term safety and efficacy study of sarilumab with or without conventional synthetic disease-modifying antirheumatic drugs (csDMARDs) in rheumatoid arthritis reported gastrointestinal perforations were rare [27], but not absent.

Overall, there were 994 cases of IL-6R inhibitors-induced GI perforation reported. For tocilizumab, the median age of patients in our study was 60 years, which was consistent with the age group (59 years, IQR 49–67) reported by a nationwide cohort study in Sweden [7]. We found that women (71.6%) were more prone to GI perforations than men (18.9%), which was similar to the result that women (81.8%) reported lower intestinal perforations in an observational study by Strangfeld et al. [14]. Similarly, middle age (≥40 years) and female sex were independent risk factors for GI perforation associated with tocilizumab use in the logistic regression analysis. Notably, the number of GI perforations has increased from 2019 to 2023, which might be related to the global outbreak of COVID-19 at the end of 2019, and tocilizumab is widely used as an anti-inflammatory drug [10]. The median time to onset of tocilizumab-associated GI perforations was 60 days (IQR 13–239), with approximately 71% of cases occurring within the first 6 months after starting tocilizumab. TTO analysis displayed a characteristic akin to early failure type, which meant a gradual reduction in the risk of GI perforation over time.

Regarding the outcomes of GI perforations induced by IL-6R inhibitors, 36.12% of patients had prolonged hospitalization (*n* = 359), followed by 23.34% of patients with disabilities, 21.83% of patients who had other serious events, and 13.98% of patients who died. GI perforation can result in serious outcomes and requires further attention. 

The largest number of tocilizumab-related GI perforations occurred in duodenal perforation with a high correlation (*n* = 312, ROR = 19.45, 95%CI 17.33–21.83). Two patients presented with duodenal perforations after tocilizumab [28,29]. An observational study reported that the adjusted HR (ref: DMARDs) of tocilizumab was 4.48 (95% CI 2.0 to 10.0) in lower intestinal perforations [14]. In a population-based study, Rempenault et al. found that tocilizumab was associated with increased odds of diverticulitis as well as GI perforation due to diverticulitis [9].

To analyze the risk of GI perforation associated with tocilizumab treatment, univariate and multivariate logistic regression analyses were conducted. The results showed that high-risk factors included female, age (≥40 years), glucocorticoids, tocilizumab, and NSAIDs. Some studies have reported risk factors for GI perforations in RA, including age, glucocorticoids, NSAIDs, Charlson Comorbidity Index (CCI), and history of diverticulitis [14,30,31]. We also found that GI perforations in combination with methotrexate (*p* = 0.326) were not significant in multivariate logistic regression analysis but were significant in univariate logistic regression. Similarly, Rempenault et al. found that only older age was a risk factor for GI perforations [9]. In a long-term safety study of tocilizumab, univariate regression analysis showed that a baseline dose of glucocorticoids increased the risk of GI perforations in univariate regression analysis [25]. Notably, the stratification and logistic regression analyses showed that the age group (<20 years) was not significant.

At present, the mechanism underlying GI perforation induced by tocilizumab is unknown. One biological mechanism is that IL-6 expression is observed early after GI injury in which IL-6 maintains small intestinal crypt homeostasis and regulates vascular endothelial growth to support epithelial proliferation and wound healing [7,9,19,20]. Another biological mechanism reported is that the IL-6 receptor plays an important role in the intestinal barrier in which locally accumulated fat tissue might cover inflamed diverticula with a high concentration of IL-6, and creeping fat may limit the deterioration of disease [14,32,33]. In some COVID-19 cases with GI perforations, COVID-related infectious colitis caused colonic ileus, resulting in GI perforations due to neuronal injury from a neuroinvasive propensity [34,35].

In recent years, the mortality rate of GI perforations among DMARD-treated patients was approximately 11.1–46%, especially with tocilizumab [7,9,14,31]. The nomogram demonstrated potential value in clinical practice which could help clinicians to predict risk or prognosis [36]. Early assessment of the risk of GI perforations is important to control patient prognosis. Therefore, we established a model to predict the risk of GI perforation after tocilizumab treatment based on six predictors identified through stepwise backward regression: sex, age, weight, glucocorticoids, IL-6R inhibitors, and NSAIDs. The ROC, calibration, and decision curves of GI perforations in the training and validation sets showed that the model had a good predictive ability and clinical applicability. These six predictors were mostly consistent with the high-risk factors reported in previous studies. This simple predictive nomogram could help clinicians quickly assess the risk of GI perforation after IL-6R inhibitors treatment.

We found that IL-6R inhibitors had a risk of GI perforations, not only tocilizumab. The risk factors of GI perforations were associated with sex, age, glucocorticoids, and NSAIDs, consistent with some studies [14,30,31]. Clinicians should pay attention to these factors in clinical practice. For example, effective intervention can be achieved in the early stage of GI perforations, like diverticulitis and gastric ulcer. For high-risk patients, we should need to monitor their gastrointestinal status.

Our study had some limitations. First, because the FAERS is a public self-reporting system, missing, duplicated, and inaccurate data are inevitable, which could lead to biases. Therefore, we could not calculate the rate of GI perforation. Although we tried some measures (excluding missing, duplicated, and inaccurate data) to reduce biases, the biases could not be eliminated. The biases might have a direct impact on ROR values, so more clinical trials or case reports are needed. Second, because other IL-6R inhibitors had few records in FAERS or were not marketed in the United States, we only included the cases of tocilizumab and sarilumab. Third, we could not clarify the causal relationship between IL-6R inhibitors and GI perforations because only the disproportion analysis was statistically significant. Fourth, because of incomplete records, we did not consider other factors in the model, such as interaction effects and comorbidities. Fifth, we used internal data to validate our prediction model, without using external data. Finally, our study included a significantly higher number of women than men, which may introduce a gender bias. More detailed variables from large sample trials are needed to refine the model in the future. Despite these limitations, we developed a nomogram based on the FAERS data to predict GI perforation after IL-6R inhibitors treatment.

## 4. Materials and Methods

### 4.1. Study Design and Data Mining 

Our pharmacovigilance study used a disproportionate analysis to assess whether there was a correlation between IL-6R inhibitors and GI perforations using the two algorithms. We extracted the American Standard Code for Information Exchange (ASCII) data from 2010 Q1 to 2024 Q1 from the FAERS database (https://fis.fda.gov/extensions/FPD-QDE-FAERS/FPD-QDE-FAERS.html, accessed on 17 June 2024). We removed duplicated data based on the unique ID (primaryid) in the demographic file [DEMO] [37]. We used the common names “tocilizumab”, “sarilumab” and their types from the Medical Subject Headings as the target drugs and selected the primary suspected (PS) as the drug role code in the drug file [DRUG]. We also excluded missing data (sex, age, weight, and reporting country) to analyze the risk of GI perforation using a logistic regression model. The study (including data identification, extraction, processing, outcomes, and exclusion criteria) is shown in Supplementary Figure S1.

### 4.2. Time-to-Onset Analysis

The TTO of adverse events was calculated using the following formula:

*Time-to-onset = Event onset date (EVENT_DT) − Therapy start date (START_DT)*

after removing reports with erroneous dates (where EVENT_DT precedes START_DT), as well as those with missing or imprecise dates [22]. TTO data characteristics were evaluated using the Weibull shape parameter (WSP) test [38]. When the shape parameter β < 1 and its 95% CI < 1, the AE is considered to decrease over time (early failure-type profile); when the shape parameter β is equal to or close to 1 and its 95% CI includes a value of 1, it indicates that the AE does not change over time (random failure-type profile); and when the shape parameter β > 1 and its 95% CI exclude a value of 1, it indicates that the AE increases over time (wear-out failure-type profile).

### 4.3. Statistical Analysis

In our study, we applied two different algorithms based on a 2 × 2 contingency table: the reporting odds ratio (ROR) and the Bayesian confidence propagation neural network (BCPNN) [39,40]. The calculation methods and criteria for the algorithms are presented in Supplementary Table S2. Among these, GI perforations conform to the criteria of the two algorithms, which are suspected to be positive signals [41]. Higher values of the algorithms indicated a stronger association between GI perforation and the target drug [42]. We also performed a stratification analysis of GI perforations. After excluding missing data, *Pearson’s* chi-square test was used to compare the differences between the groups [43]. Our study used univariate and multivariate logistic regression analyses to assess risk factors for GI perforations [44]. We used two methods to calculate the minimal sample size required, including a 20× events per variable criterion (EPV) and a pmsampsize package (R software, version 4.4.1) [45,46]. The minimal sample size required was 2000 cases with 100 events or 3757 cases with 22 events. Finally, we included 9717 cases with 360 GIPs-positive events in a model for predicting the risk of GI perforation caused by IL-6R inhibitors after excluding missing data. The area under curve (AUC) value, which indicates a discriminant ability ranging from 0.5 (random chance) to 1.0 (perfect fit), suggests a reasonable estimation when it exceeds 0.7. SAS software (version9.4), Zstats 1.0 (www.zstats.net, accessed on 13 July 2024), and R software (version 4.4.1) were used for the statistical analysis.

## 5. Conclusions

Our study applied different methods to analyze the correlation between IL-6R inhibitors and GI perforations, including disproportionation analysis, regression analysis, chi-square test, time-to-onset analysis, and prediction model. We found that the following factors might increase the risk of GI perforation: female, ≥40 years, high body weight, IL-6R inhibitors, glucocorticoids, and NSAIDs. Most reported GI perforations (71%) occurred within the first six months after tocilizumab treatment. Therefore, we suggested that the risk of gastrointestinal perforations should require more attention based on the predictive nomogram, especially for older patients with IL-6R inhibitors, glucocorticoids, and NSAIDs.

## Figures and Tables

**Table 1:** The signal strength of gastrointestinal perforations associated with IL-6R inhibitors from the FAERS database.

Preferred Terms (PTs)	Tocilizumab			Sarilumab			*p*
	Cases	ROR	IC (IC_025_)	Cases	ROR	IC (IC_025_)	
Duodenal perforation	312 *	19.45 (17.33–21.83)	4.18 (3.72 ^a^)	14 *	9.57 (5.66–16.17)	3.25 (1.92 ^b^)	0.18
Intestinal perforation	176 *	4.63 (3.99–5.37)	2.19 (1.89 ^b^)	2	-	0.86 (0.02)	-
Gastrointestinal perforation	150 *	10.76 (9.14–12.68)	3.37 (2.86 ^b^)	2	-	2.21 (0.18)	-
Large intestine perforation	136 *	3.95 (3.33–4.68)	1.96 (1.66 ^b^)	7 *	4.49 (2.14–9.43)	2.16 (1.03)	0.62
Diverticular perforation	101 *	12.72 (10.41–15.54)	3.60 (2.95 ^b^)	3 *	7.63 (2.46–23.71)	2.93 (0.94)	0.23
Small intestinal perforation	42 *	4.03 (2.97–5.47)	1.99 (1.47)	1	-	-	-
Gastric perforation	29 *	2.12 (1.47–3.05)	1.08 (0.75)	1	-	-	-
Perforation	9	-	0.79 (0.41)	-	-	-	-
Upper gastrointestinal perforation	3 *	22.98 (7.04–75.05)	4.40 (1.35)	-	-	-	-
Procedural intestinal perforation	2	-	1.14 (0.28)	1	-	-	-
Lower gastrointestinal perforation	3 *	16.71 (5.19–53.83)	3.97 (1.23)	-	-	-	-
Total	963 *	6.86 (6.43–7.31)	2.74 (2.57 ^b^)	31 *	4.03 (2.83–5.73)	2.00 (1.41)	<0.001

* showed statistically significant signals by two algorithms (ROR and IC_025_, case > 2)—showed non-significant signals. *p* used the Pearson chi-square test. ^a^: IC_025_ > 3.0, it indicates a strong signal; ^b^: 1.5 < IC_025_ ≤ 3.0, it indicates a medium intensity signal. ROR, reporting odds ratio; CI, confidence interval; IC, information component; IC_025_, the lower limit of 95% CI of the IC.

**Table 2:** Risk factors of gastrointestinal perforations included by IL-6R inhibitors from the FAERS database.

Variables	Univariate Logistic					Multivariate Logistic				
	β	S.E	Z	*p*	OR (95%CI)	β	S.E	Z	*p*	OR (95%CI)
**Sex**										
Male					1.00 (Reference)					1.00 (Reference)
Female	0.37	0.13	2.79	0.005	1.45 (1.12–1.88)	0.42	0.14	3.08	0.002	1.52 (1.16–1.98)
**Age, years**										
0–19					1.00 (Reference)					1.00 (Reference)
20–39	0.58	0.68	0.85	0.394	1.78 (0.47–6.75)	0.31	0.68	0.46	0.647	1.37 (0.36–5.23)
≥40	2.03	0.58	3.49	<0.001	7.59 (2.43–23.72)	1.73	0.59	2.94	0.003	5.63 (1.78–17.78)
**Weight, kg**	0.01	0.00	4.38	<0.001	1.01 (1.01–1.01)	0.01	0.00	3.67	<0.001	1.01 (1.01–1.01)
**Glucocorticoids**										
No					1.00 (Reference)					1.00 (Reference)
Yes	0.55	0.11	5.09	<0.001	1.74 (1.40–2.15)	0.32	0.11	2.81	0.005	1.37 (1.10–1.72)
**NSAIDs**										
No					1.00 (Reference)					1.00 (Reference)
Yes	1.31	0.11	12.03	<0.001	3.70 (2.99–4.57)	1.24	0.11	10.96	<0.001	3.46 (2.77–4.32)
**Methotrexate**										
No					1.00 (Reference)					1.00 (Reference)
Yes	0.48	0.11	4.37	<0.001	1.62 (1.31–2.02)	0.12	0.12	0.98	0.326	1.12 (0.89–1.42)
**IL-6R inhibitor**										
Sarilumab					1.00 (Reference)					1.00 (Reference)
Tocilizumab	0.67	0.34	1.96	0.050	1.95 (1.01–3.81)	1.08	0.35	3.13	0.002	2.94 (1.50–5.79)

FAERS: Food and drug administration adverse event reporting database. OR: Odds ratio. CI: Confidence interval. NSAIDs: nonsteroidal anti-inflammatory drugs. Weight (>100 kg) may be biased due to less cases.

## Data Availability

The datasets generated and analyzed during the current study are available from the corresponding author upon reasonable request. The FAERS database is available at https://fis.fda.gov/extensions/FPD-QDE-FAERS/FPD-QDE-FAERS.html, accessed on 17 June 2024.

## Supplementary Materials

The following supporting information can be downloaded at: https://www.mdpi.com/article/10.3390/biomedicines12122860/s1, Figure S1: The flow diagram of the study about the gastrointestinal perforations associated with IL-6R inhibitors. Table S1: The results of time-to-onset analysis for signals with gastrointestinal perforations of IL-6R inhibitors. Table S2: Summary of major algorithms used for signal detection.
