# Supplementary material for: IL-6R Inhibitors and Gastrointestinal Perforations: A Pharmacovigilance Study and a Predicting Nomogram
Source: Biomedicines. 2024 Dec 17;12(12):2860. doi: 10.3390/biomedicines12122860 (PMC11673817; doi:10.3390/biomedicines12122860)
Supplement: Supplementary file 1 [file biomedicines-12-02860-s001.zip › biomedicines-3328877-supplementary.pdf]

Supplementary Materials

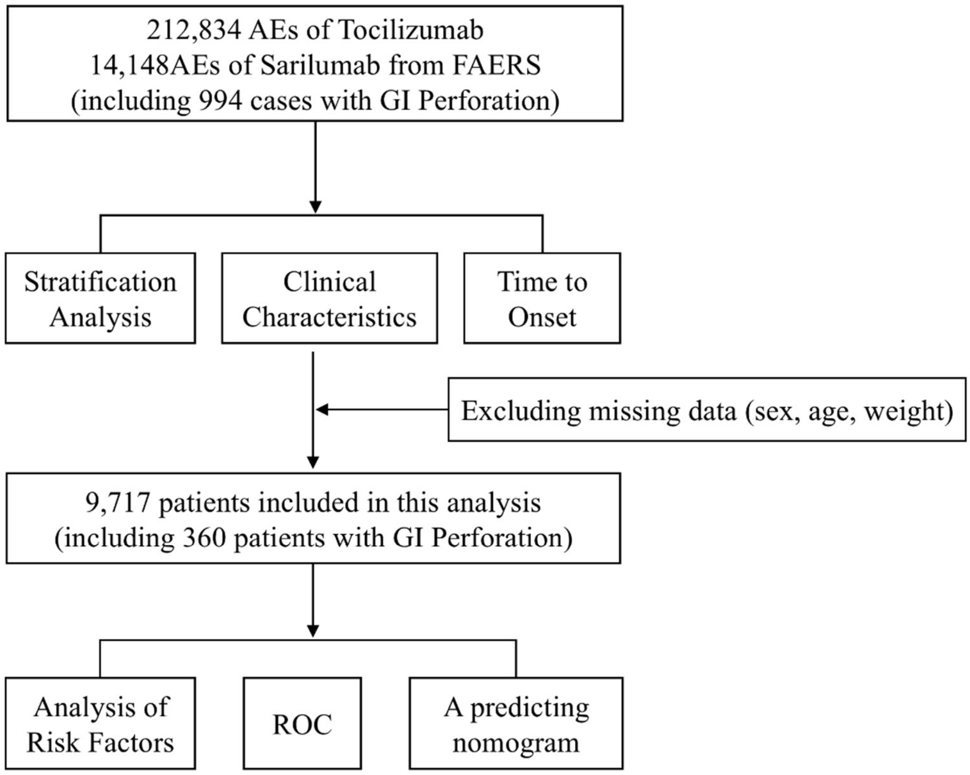

Supplementary Figure S1. The flow diagram of the study about the gastrointestinal perforations associated with IL-6R inhibitors.
GI: gastrointestinal. ROC: receiver operating characteristic.

Supplementary Table S1. The results of time-to-onset analysis for signals with gastrointestinal perforations of IL-6R inhibitors.

IL-6R inhibitors	TTO (days)			Weibull distribution				Failure type
	Cases	Median (IQR)	Min–max	Scale parameter		Shape parameter		
	n			α	95% CI	β	95% CI	
**Tocilizumab**	218	60 (13-239)	0-728	130.47	104.24-156.69	0.72	0.64-0.80	Early Failure
**Sarilumab**	13	79 (13-202)	0-435	142.94	52.34-233.54	0.98	0.51-1.45	Random failure

n, number of cases with available time-to-onset; IQR, interquartile range; TTO, time-to-onset. When TTO is 0 days, the adverse event occurred within the same day with the therapy.

Supplementary Table S2. Summary of major algorithms used for signal detection.

Algorithms	Equation	Criteria
ROR	ROR=ad/bc<br>$95\%CI=e^{\ln(ROR)\pm1.96(1/a+1/b+1/c+1/d)^{\wedge}0.5}$	lower limit of 95% CI>1, N≥3
BCPNN	$IC=\log_2 a(a+b+c+d)/((a+c)(a+b))$<br>$IC_{025}=e^{\ln(IC)-1.96(1/a+1/b+1/c+1/d)^{\wedge}0.5}$	$IC_{025}>0$

a: the number of reports with suspect adverse events (ADEs) of the suspect drug; b: the number of reports with all other ADEs of the suspect drug; c: the number of reports with the suspect ADE of all other drugs; d: the number of reports with all other ADEs of all other drugs; ROR: reporting odds ratio; CI: confidence interval; N: the number of co-occurrences; BCPNN: Bayesian confidence propagation neural network; IC: information component; IC_{025}: the lower limit of the 95% two-sided CI of the IC.
